# INSL3 in the Ruminant: A Powerful Indicator of Gender- and Genetic-Specific Feto-Maternal Dialogue

**DOI:** 10.1371/journal.pone.0019821

**Published:** 2011-05-16

**Authors:** Ravinder Anand-Ivell, Stefan Hiendleder, Carolina Viñoles, Graeme B. Martin, Carolyn Fitzsimmons, Andrea Eurich, Bettina Hafen, Richard Ivell

**Affiliations:** 1 Sansom Institute, and School of Pharmacy and Medical Sciences, University of South Australia, Adelaide, South Australia, Australia; 2 School of Medical Sciences, University of Adelaide, South Australia, Adelaide, Australia; 3 Robinson Institute and School of Animal and Veterinary Sciences, University of Adelaide, Adelaide, South Australia, Australia; 4 School of Animal Biology M092, Faculty of Natural and Agricultural Sciences, University of Western Australia, Crawley, Western Australia, Australia; 5 Robinson Institute and School of Molecular and Biomedical Science, University of Adelaide, Adelaide, South Australia, Australia; University of Córdoba, Spain

## Abstract

The hormone Insulin-like peptide 3 (INSL3) is a major secretory product of the Leydig cells from both fetal and adult testes. Consequently, it is a major gender-specific circulating hormone in the male fetus, where it is responsible for the first phase of testicular descent, and in the adult male. In most female mammals, circulating levels are very low, corresponding to only a small production of INSL3 by the mature ovaries. Female ruminants are exceptional in exhibiting high INSL3 gene expression by the thecal cells of antral follicles and by the corpora lutea. We have developed a specific and sensitive immunoassay to measure ruminant INSL3 and show that, corresponding to the high ovarian gene expression, non-pregnant adult female sheep and cows have up to four times the levels observed in other female mammals. Significantly, this level declines during mid-pregnancy in cows carrying a female fetus, in which INSL3 is undetectable. However, in cows carrying a male fetus, circulating maternal INSL3 becomes elevated further, presumably due to the transplacental transfer of fetal INSL3 into the maternal circulation. Within male fetal blood, INSL3 is high in mid-pregnancy (day 153) corresponding to the first transabdominal phase of testicular descent, and shows a marked dependence on paternal genetics, with pure bred or hybrid male fetuses of *Bos taurus* (Angus) paternal genome having 30% higher INSL3 levels than those of *Bos indicus* (Brahman) paternity. Thus INSL3 provides the first example of a gender-specific fetal hormone with the potential to influence both placental and maternal physiology.

## Introduction

The peptide hormone Insulin-Like Peptide 3 (INSL3; formerly relaxin-like factor, RLF) belongs to the relaxin-insulin family of peptide hormones [1], [2]. It evolved as a paralogue of relaxin accompanying mammalian emergence [3], and like relaxin appears to subserve “neohormone” functions [4], the most important of which is to regulate the first transabdominal phase of testicular descent in the embryo during mid-gestation [2]. It is produced in large quantities by the Leydig cells of both the fetal and adult testes, and gives rise to substantial circulating INSL3 concentrations in the blood of adult male mammals (rat, 5 ng/ml [5]; mouse, 2 ng/ml [5]; human, 0.8–2.5 ng/ml [6]–[8]; rhesus monkey, 1.5 ng/ml (unpublished)). To date there is very little information about INSL3 peptide levels within the fetus. We have shown in human pregnancies that amniotic fluid contains substantial amounts of INSL3 of fetal origin, which can only be detected in male fetuses and has its maximum at weeks 12–16 of gestation at the time of the transabdominal phase of testicular descent [9]. In preliminary studies in rats, we have also shown that male fetuses in the second half of gestation have similar amniotic INSL3 concentrations to those measured in human amniotic fluid, and that blood from such male fetuses contained INSL3 concentrations comparable to adult males (Ivell, Anand-Ivell & Barthol, unpublished). In all cases INSL3 was below the level of detection in fluids from female fetuses.

In the adult female mammal circulating INSL3 concentrations are much lower than in the male (rat, 0.08 ng/ml [5], mouse, 0.05 ng/ml [5]; human, 0.05–0.10 ng/ml [6], [7], [10]), and presumably reflect local production of INSL3 within the ovary [2]. Here immunohistochemical and mRNA evidence supports a production by the theca layer of smaller antral follicles, as well as by corpora lutea [11]–[14]. In fact, women with polycystic ovarian syndrome are found to have almost double the normal circulating levels of INSL3 [10], [15], which appears to be associated with the number of cystic follicles [15].

Within this context ruminants appear to be special, with the ovaries expressing very high levels of INSL3 mRNA both in antral follicles and in the corpus luteum [11], [16]. It has been speculated that in some way the high INSL3 expression might be compensating for the fact that in ruminant evolution the closely related gene for relaxin has been lost [11], though there is as yet no functional evidence to support this idea. As in other species, INSL3 mRNA is expressed by the theca interna cells of antral follicles and appears to be negatively regulated by high LH [16], although it is also expressed after luteinisation within the corpus luteum. Across the estrous cycle, luteal INSL3 mRNA levels rise from early to mid cycle and then decline again at luteolysis unless pregnancy occurs, when INSL3 mRNA continues to rise until mid gestation and remains elevated until shortly before birth [16].

Although peptide INSL3 has been successfully extracted from bovine testis [17], to date there is no information about INSL3 levels in the circulation of any ruminant, especially within females, which might offer clues to the high level of expression in the ovaries of sheep and cows. We have successfully developed a new time-resolved fluorescence immunoassay (TRFIA) to directly detect INSL3 in the blood and body fluids of ruminants. We have used this new assay to measure circulating INSL3 firstly across the estrous cycle in synchronized gravid and non-gravid sheep, and secondly in both maternal and fetal bloods through gestation from a controlled experiment with *Bos taurus* and *B.indicus* cows. Not only are female circulating INSL3 concentrations significantly higher than in other non-ruminant species, but during gestation we see for the first time for any species and hormonal factor, clear evidence of a fetal gender-specific elevation in maternal serum INSL3, most likely derived by placental transfer of INSL3 from the male fetus to the mother. In addition, our bovine model with purebred and reciprocal hybrid fetuses shows a highly significant effect of fetal genetics on fetal INSL3 production, which is not simply reflecting the known influences of heterosis on fetal growth.

## Results

### INSL3 concentration during the estrous cycle and early pregnancy in ewes

**Figure 1:**
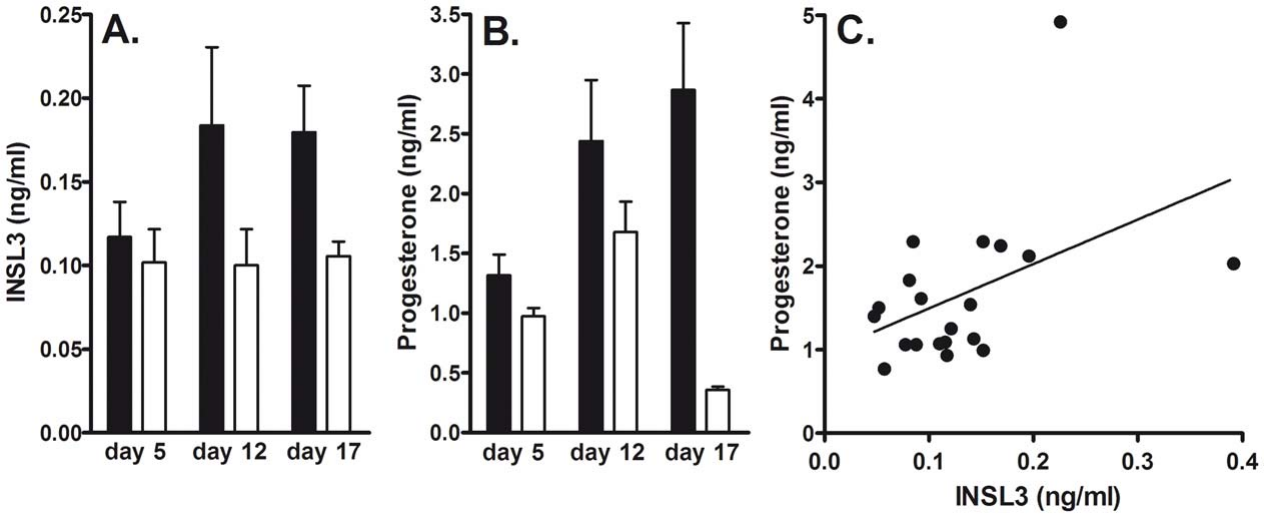
Circulating INSL3 (A) and progesterone (B) concentrations in gravid (filled bars; N = 6) and non-gravid (open bars; N = 4) sheep (means ± SD). Statistical significance is shown only where P<0.05. (C) Correlation (r = 0.4; P<0.05) of INSL3 and progesterone values for samples collected on days 5 and 12 only, excluding day 17 (luteolysis).

Blood samples were systematically collected at three time points (days 5, 12, and 17) from the 17-day cycle of 6 gravid and 4 non-gravid synchronized ewes. No significant differences in circulating INSL3 concentration were observed for any day between gravid and non-gravid sheep (Fig.1A), although in the pregnant ewes the increase in INSL3 concentrations on day 12 compared to day 5 approached significance (P = 0.06), and values were close to being significantly different between gravid and non-gravid animals (P = 0.07) on day 17. Peptide levels appear to reflect what is known of the luteal INSL3 mRNA expression with levels generally elevated from day 5 through to day 17, especially in the gravid cycles. The comparable circulating progesterone concentrations are indicated in Fig.1B, where the expected significant difference at luteolysis can be observed between gravid and non-gravid ewes. A comparison of all INSL3 values from days 5 and 12 (thus excluding those undergoing luteolysis or not at day 17) against the corresponding levels of circulating progesterone (Fig.1C) showed a significantly positive relationship (P<0.01) reinforcing the luteal origin of the circulating INSL3. It is to be noted that whereas all INSL3 values from gravid animals showed a consistent 15 to 223% increase between days 5 and 12, corresponding values from the 4 non-gravid animals were more variable, ranging from −53% to +73%.

### Circulating INSL3 concentrations during pregnancy in cows

**Figure 2:**
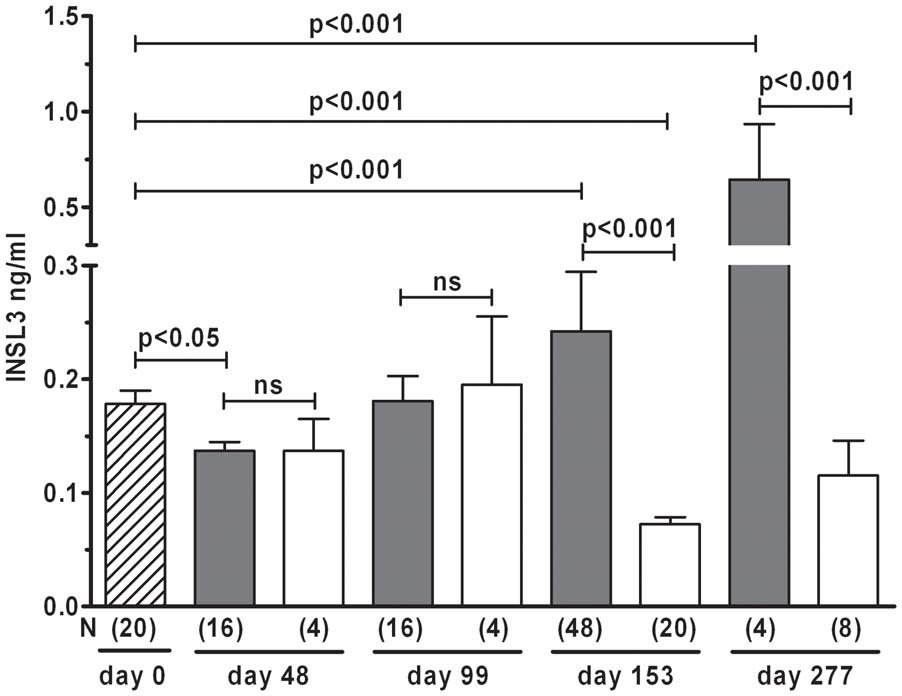
Circulating maternal concentrations of INSL3 in pregnant Brahman and Angus cows (combined since no significant difference between breeds at any time-point). Filled bars represent cows carrying a male fetus, open bars those carrying a female fetus. The cross-hatched bar (left) represents cows at the time-point of insemination. N values are indicated in parentheses below the bars. Statistical significance using both t test and Neumann-Keuls post hoc test are as indicated (ns, not significant).

Blood samples were collected from heifers of the Angus (*B.taurus*) and Brahman (*B.indicus*) breeds at the time of synchronized insemination (day 0) and were considered representative of the non-gravid cow. There was no significant difference in peripheral INSL3 concentration (mean ± SEM) between the two breeds (Angus: 0.17 ± 0.02 ng/ml, n = 10; Brahman: 0.18±0.01 ng/ml, n = 10), with values very similar to those observed in sheep (Fig.1A). This lack of maternal breed difference did not change throughout gestation, and thus in subsequent analyses maternal genotype is combined (Fig.2). Plotting maternal INSL3 concentrations across gestation showed little variation, unless fetal gender is taken into account (Fig.2). Following an initial small decline in circulating maternal INSL3 (p<0.05 for animals carrying a male fetus), levels remained around the day 0 values until the end of the first trimester (day 99). At mid-gestation (day 153), cows carrying a male fetus showed significantly (p<0.001) higher maternal INSL3 as compared with cows carrying a female fetus, where maternal INSL3 concentrations declined to levels significantly below day 0 values (p<0.01). Consequently, at day 153, maternal INSL3 concentration clearly distinguishes the gender of the fetus (p<0.001). This gender-specific effect on maternal INSL3 levels appears to be maintained until term (day 277). Because of the relatively high variance in maternal INSL3 concentration, we were unable to detect any effect of fetal genetics in those cows carrying male fetuses (not shown).

### Fetal INSL3 concentrations, gender and genetics

**Figure 3:**
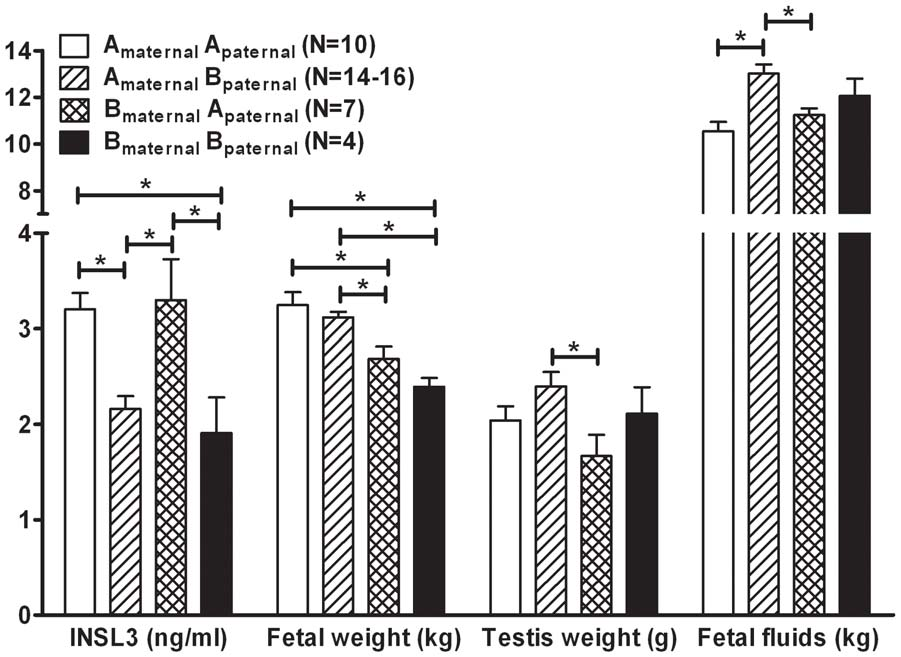
INSL3 concentration in fetal venous blood, fetal weight, fetal testis weight, and calculated total fetal fluids on day 153 of pregnancy, and sorted by fetal genetics, as indicated. A_maternal_ indicates an Angus (*B. taurus*) dam, B_paternal_ a Brahman (*B. indicus*) sire, etc.

In addition to maternal bloods, we also analysed fetal blood from day 153 pregnancies for INSL3 concentration. Without exception, all female fetuses had undetectable (<0.02 ng/ml) levels of INSL3 (data not shown). This was also true for the small number of female fetuses analysed at term (day 277). In contrast all male fetuses indicated substantial circulating concentrations of INSL3 (range 1–5 ng/ml). There was a pronounced effect of fetal genetics on the circulating concentration of INSL3 within these male fetuses (Fig.3). Purebred Angus fetuses as well as the Brahman_maternal_ x Angus_paternal_ hybrids had significantly higher serum INSL3 concentrations compared to either purebred Brahman or Angus_maternal_ x Brahman_paternal_ hybrids (Fig.3). The 30 percent increase in the INSL3 plasma concentration of fetuses sired by Angus bulls indicates a strong paternal genetic effect on INSL3 in the fetus. It should be noted that we found no significant correlation between fetal INSL3 and maternal INSL3 concentrations in cows carrying a male fetus (data not shown).

Heterosis or hybrid vigour is a well established biological phenomenon where the phenotype of F_1_ hybrids is superior to the parental phenotype. In the present study, there was no obvious heterosis effect in terms of fetal weight at day 153 (Fig.3), with purebred Angus and Angus_maternal_ x Brahman_paternal_ fetuses being significantly heavier than the Brahman_maternal_ x Angus_paternal_ and purebred Brahman fetuses. In fact, the Brahman_maternal_ x Angus_paternal_ hybrid was intermediate in weight between the two purebred parental breeds. Since INSL3 appears to be uniquely a product of the fetal testis, we investigated if the genetic effects on fetal INSL3 concentration could be due to altered growth and development of the fetal testes. However, examination of fetal testis weights showed the opposite trend, with fetuses of purebred genetics having similar testis weights, and the reciprocal hybrids having heavier (Angus_maternal_ x Brahman_paternal_) or lighter (Brahman_maternal_ x Angus_paternal_) testes than both purebreds, although only the differences between hybrids were statistically significant (Fig.3). Thus neither fetal weight nor testis weight can account for the marked differences in INSL3 concentrations of fetuses with different genetics. A further factor that might contribute to the differences in fetal INSL3 concentration is the amniotic fluid volume. We have previously shown that early second trimester human amniotic fluid contains substantial concentrations of INSL3 [9] presumably derived from the fetal circulation at a time when fetal vasculature and tissues are still relatively permeable. Fetal INSL3 concentration might then reflect a dilution effect caused by a genetics-dependent difference in amniotic fluid volumes and/or fetal weight. However, the actual data again show that this is not the case (Fig.3). There is no clear inverse relationship of gestational volume with fetal INSL3 concentration, and even though there is an apparent inverse relationship between INSL3 levels and total amount of fetal fluid volume, the genotype-dependent differences in the latter are too small to account for the large changes in fetal serum INSL3 concentration.

## Discussion

Previous studies at the mRNA level have indicated that within the female ruminant the ovary represents by far the major source of INSL3 [11], [16]. Northern blot analysis indicates very strong signals in the corpus luteum of the cycle and pregnancy, with similar strong signals also deriving from primary preparations of follicular theca cells. By weight, the corpora lutea are likely to be the major ovarian source of INSL3 peptide, rather than the theca interna, although to date this has not yet been analysed directly. All other tissues examined, including oviduct, endometrium and myometrium from the cycle and pregnancy, caruncles, cotyledons, amnion and chorion, all from term pregnancies, thyroid gland, hypothalamus, heart, lung, cerebellum, cerebral cortex, pituitary and pineal glands, spleen, liver and adrenal gland were all negative for INSL3 mRNA by northern analysis, with only very weak signals appearing sporadically from some tissues following the use of RT-PCR and product visualization by radioactive hybridization, which is a highly sensitive detection technique [16]. Reflecting this expression pattern, we see high levels of circulating INSL3 (ca. 0.1 to 0.2 ng/ml) throughout the estrous cycle in both sheep and cows. This is approximately twice to four times the circulating levels seen in healthy women [10], [15]. Interestingly, if the INSL3 concentrations from days 12 and 17 are combined, then these do show a significant difference (P<0.05) between gravid and non-gravid ewes. Combining INSL3 and progesterone values for individual ewes on day 17 provided a pregnancy index with 100% positive and 100% negative predictive value. This now needs to be validated for a larger sample size.

In cows, non-pregnant circulating INSL3 concentrations appear to be maintained during the first trimester of pregnancy, with possibly a small decline at day 48. In dams carrying female fetuses, which do not themselves express INSL3, the circulating maternal INSL3 concentration then declines to low values (ca. 0.1 ng/ml) by mid-gestation, which appear to be maintained until term. This is of interest since our earlier studies had shown that luteal levels of mRNA are maximal at this time [16]. This would suggest that other post-transcriptional mechanisms such as peptide production, secretion rates, serum half-life or luteal blood flow are regulating the peripheral INSL3 concentration. It is well established that, in terms of oxytocin and progesterone production, the bovine corpus luteum has markedly reduced influence in the latter half of gestation (except possibly at term), when these functions are largely taken over by the pituitary and placenta, respectively [18].

This situation is quite different for dams carrying a male fetus. For the first time for any truly fetal hormone (excluding trophoblast-derived hCG or interferon-tau), we can detect significantly increased levels of INSL3 in maternal serum at mid-pregnancy (day 153), which must be derived uniquely from the male fetus. From the results on female fetal blood, as well as earlier results on human amniotic fluid [9], we know that there is no detectable contribution of INSL3 to maternal serum from the female fetus, nor from the placenta. The results presented here suggest that the male fetus is contributing ca. 0.2 ng/ml INSL3 to the maternal circulation, and hence also to all maternal organs including the *Placenta materna*. This fetal signal appears to be maintained throughout the latter half of gestation. Whilst there is always the possibility that this gender-specific rise in maternal INSL3 could be caused by an indirect fetal effect on the ovary, this appears highly unlikely in view of the obvious lack of any other gender-specific fetal factor which might stimulate this effect, and the much more obvious cause of a trans-placental transfer of INSL3 directly from the male fetus.

Direct measurement of INSL3 in mid-term fetal blood indicates that the male fetus is producing large amounts (range 1–5 ng/ml) of the gonadal peptide into the fetal circulation at this time, concomitant with its role to stimulate gubernacular development and the first transabdominal phase of testicular descent [19]. Surprisingly, there is a marked effect of fetal genetics on fetal INSL3 production, with the paternal genetics determining INSL3 levels. Fetuses with *Bos taurus* (Angus) paternal genetics show approximately 30% higher INSL3 levels than fetuses with *B. indicus* (Brahman) paternal genetics. We have previously noted marked genetic effects on adult INSL3 concentrations amongst different laboratory rat strains [5]. The effect of paternal genetics on bovine fetal INSL3 production is independent of testis size, as well as of fetal size and the amount of fetal fluid, and thus does not appear to be an aspect of any heterosis effects in this experiment. This is consistent with preliminary analysis of pregnancy parameters [20] which revealed that at mid-gestation (day 153) hybrids did not show heterosis in fetal size, but were intermediate to both parental genetics. Taken together, these results imply that paternal genetics is determining the level of INSL3 independently of the relative sizes of fetal testis, total fetal weight or amount of fetal fluid. It is noteworthy in this context that the Y chromosomes of *Bos taurus* (e.g. Angus) and *Bos indicus* (e.g. Brahman) show considerable structural and size differences [21] and that Y chromosome substitution in model organisms has revealed effects on the expression of thousands of genes, presumably mediated by epigenetic effects of transposons [22]. The current experiments did not involve embryo transfer, and so we are not able to analyse what effects might be observed when pure-bred fetuses (e.g. Brahman_maternal_ × Brahman_paternal_) develop in the alternative maternal genotype (e.g. Angus_maternal_ × Angus_paternal_), though it might be difficult here to distinguish effects of maternal genotype from those consequent upon an IVF protocol [23].

There is some evidence to suggest that there may be a breed difference in cattle in the incidence of cryptorchidism [24]. Though Brahman cattle were not included in this study, it is therefore possible that bulls of different breeds may have differing levels of INSL3 production, as in rats [5]. A study to examine this aspect of INSL3 physiology is planned.

The results presented here imply that INSL3 from the male fetus is able to cross the placenta and enter the maternal bloodstream. Whilst there is limited evidence supporting the possible expression of INSL3 itself within the cytotrophoblast in women [25], deer [26] and dogs [27], this is evidently not of sufficient amount to influence the maternal circulation in ruminant pregnancies carrying a female fetus. Conversely, it is interesting to note that in cows carrying a female fetus the maternal INSL3 contribution, presumably of ovarian origin, significantly (p<0.002) declines between days 99 and 153. This could be interpreted as indicating that the maternal INSL3 observed in the non-pregnant cow might otherwise also be able to cross the placenta in the direction of the fetus, where it could induce some kind of ovarian descent or other malfunction, as has been demonstrated in female mouse fetuses transgenically expressing the Insl3 gene [28]. There are still too few data available to know whether such placental transport would be a general feature in mammals, or whether it is a special property of the ruminant placenta.

Since maternal circulating INSL3 remains unchanged in pregnant women, whether carrying a male or a female fetus (Anand-Ivell & Ivell, unpublished), it would appear most likely that any more general mammalian function for fetal INSL3 is likely to be confined to the feto-placental unit. INSL3 has been shown to be the unique ligand in the low nanomolar range of the RXFP2 receptor, previously called LGR8 [29], [30]. It has also been shown in vitro to be able to activate the receptor, RXFP3, for the neuropeptide relaxin-3, but only at very high, micromolar concentrations [31]. Thus in order to discuss what role a fetal gender-specific hormone like INSL3 is having during gestation, beyond its known role in testicular descent [2], we need to examine where RXFP2 receptors are expressed within the uterus and placenta. In the human and rat, RXFP2 appears to be expressed only in the myometrial layers [32], [33] but not within any placental tissue [34]. Within human myometrial cells from the menstrual cycle, RXFP2 was shown not to respond in a typical Gs- or Gi-linked fashion [33] suggesting that its role is likely to be more subtle. However, Vodstrcil and colleagues [32] were able to show that myometrial RXFP2 receptor mRNA was up-regulated in rat gestation by an IUGR intervention, supporting a role for this INSL3-RXFP2 system in the feto-placental dialogue. Furthermore, in women undergoing first trimester amniocentesis and later diagnosed with preeclampsia [9], INSL3 concentrations in amniotic fluid were significantly elevated compared to normal controls at a time when the placenta was evidently not developing correctly, even though preeclamptic symptoms would first become evident months later. In pregnant cows at least this gender-specific elevation of circulating INSL3 is likely to have a positive effect on bone density, reflecting the recent discovery in humans and mice that the INSL3-RXFP2 system is significantly involved in bone metabolism [35].

For humans, there is some evidence for fetal gender-specific effects on the incidence of preterm labour and preeclampsia (both more likely for male fetuses; [36], [37]), whereas IUGR appears to be marginally more likely for female fetuses [37]. Such data are, however, controversial [38]. It is also reported that there is a fetal gender-specific influence on maternal hCG (increased when carrying a female fetus) and MSAFP (decreased when carrying a female fetus) [39]. Such findings would support an influence by a male- or female- specific fetal factor on placental function. Since fetal steroids do not vary sufficiently between genders to satisfactorily account for such changes, INSL3 would present itself as a likely candidate in the feto-placental dialogue. More research is now required to explore this possibility.

In conclusion, therefore, we have shown that ruminants express moderately high levels of circulating INSL3 throughout the estrous cycle and pregnancy, but that this level increases significantly during mid-gestation only when the cow is carrying a male fetus.

## Materials and Methods

### Sheep

We used 10 Merino ewes aged 2.5 years and weighing 52.3±1.5 kg with a condition score of 2.8±0.1 (scale from 0: emaciated to 5: obese [40]) that were selected from a larger study. All ewes grazed dry summer pasture predominantly comprising barley grass and capeweed without a supplement. Sponges containing medroxy-progesterone (Cronogest®, Intervet, Australia) were inserted for 14 days and 200 IU of eCG (Folligon®, Intervet, Australia) injected at sponge removal, two days before artifical insemination (AI, Day 0). Ewes were housed in a shed without food and water for 24 h before AI, performed 49–57 hours after sponge removal. Semen from Texel rams that had been evaluated (mass motility >4; density >4) and pooled, was used for AI. The ejaculates were diluted 1∶3 with UHT skim milk and the insemination volume was adjusted to give a dose of 200 million sperm per ewe. Blood was sampled after 12 h fasting on Days 5 (day of embryo arrival in the uterus), 12 (around maternal recognition of pregnancy) and 17 (follicular phase in non-pregnant ewes) after AI. Blood plasma was harvested and stored at −20°C until assayed for progesterone and INSL3. Ovulation rate and pregnancy were diagnosed by transrectal ultrasonography using a 7.5 MHz linear array transducer on Days 10 and 30. Only ewes that had a single ovulation were selected for this study. One ewe had high a progesterone concentration on day 17, but later gave no positive signs of pregnancy at ultrasound on day 30, indicating early pregnancy loss. For this study this animal is included as pregnant. Experimental procedures were approved by the Animal Ethics Committee of the University of Western Australia (no. RA/3/100/534), according to the recommendations of the Australian National Health & Medical Research Council.

Plasma progesterone was measured in duplicate using a standard kit (Diagnostic Systems Laboratories Inc, Webster, TX) as described elsewhere [41]. The limit of detection was 0.4 ng/mL. For low (0.9 ng/mL) and high (10.4 ng/mL) progesterone concentrations, the intra-assay coefficients of variation were 2.6% and 4.1%, and inter-assay coefficients of variation 5.7% and 11.1%.

### Cattle and fetuses

We used animals of the Angus and Brahman breeds to study maternal and fetal INSL3 levels during gestation. The two breeds represent the genetics of two subspecies with taurine (*Bos primigenius taurus*) and zebuine (*B. p. indicus*) phenotype, respectively, found in domestic cow and commonly referred to as *Bos taurus* and *Bos indicus* [42]. Angus and Brahman females which had not given birth previously and were approximately 16–20 months of age received standard commercial estrous cycle synchronization (e.g. http://www.absglobal.com/aus/resources/beef-resources/synchronization-programs---cue-mate-1/). We used Cidirol - Heat Detection & Timed Insemination (HTI) and Cidirol - Timed Insemination (TI). Briefly this consisted of an initial injection of 1 ml of 1 mg/ml estradiol benzoate (Cidirol, Genetics Australia Co-operative Ltd., Bacchus Marsh, Australia) and insertion of a progesterone-releasing vaginal insert (Eazi-Breed CIDR, DEC International, Hamilton, New Zealand). The vaginal inserts were removed after 7–9 days and heifers injected with 2 ml of a prostaglandin analogue (0.26 mg of cloprostenol sodium/ml (Estrumate), Schering-Plough Animal Health, Baulkam Hills, Australia). Estrus detection devices (Kamar, Agrigene, Wangaratta, Australia) were placed on all animals. In HTI, animals that showed estrus two days later were inseminated while animals not in estrus received an additional 0. ml injection of estradiol benzoate and were inseminated 24 h later. In TI, animals received 0.7 ml estradiol benzoate the day after removal of vaginal inserts and were inseminated 24 h later. Synchronization/insemination was repeated in HTI and TI with estradiol benzoate injection of all animals after removal of vaginal inserts, followed by a final round of insemination and natural breeding in HTI animals without further synchronization measures. We used Angus and Brahman paternal genetics in HTI and TI. Animals were pregnancy tested by ultrasound scanning.

Blood samples were obtained on Days 0, 48, 99, 153 and 277 of gestation. Day 277 was considered term as gestation length in the cow ranges from Day 273 to Day 292 [43]. Fetal blood samples were collected from the umbilical cord (Day 153) after removal of the fetus from the uterus in an abattoir, or by jugular vein puncture (Day 277) after delivery of the calf by caesarian section. Serum was stored frozen at −80°C until INSL3 analysis. The amount of fetal fluids was estimated by subtracting the combined fetal, placental, and washed uterine weights from the weights of the intact gravid uteri prior to dissection. Gender of Day 48 concepti was determined by SRY typing using PCR with S4B primers [44], and of Day 99 fetuses by ultrasound scanning. All procedures involving cattle in this study were approved by The University of Adelaide Animal Ethics Committee (nos. S-094-2005 and -2005A).

### INSL3 Assay development, validation and characteristics

Bovine INSL3 was generously synthesized and made available by Dr John Wade of the Florey Institute, University of Melbourne, according to the sequence A-chain: ATAINPARHCCLSGCTRQDLLTLCPH, B-chain: QEAPEKLCGHHFVRALVRLCGGPRWSSEEDG, predicted from the cloned cDNA sequence [11]. This is essentially confirmed at the peptide level by Bullesbach & Schwabe [17] who, however, also identified molecules with a further extension at the C-terminus of the B-chain. NMR was used to confirm that the A–B heterodimer was in the correct native conformation (Dr J.D. Wade, personal communication). Polyclonal antibodies were raised in rabbits against this A–B heterodimer using conventional techniques and used to generate a robust time-resolved fluorescence immunoassay (TRFIA) exactly as previously described for the human INSL3 assay [7], [8]. As tracer for the assay, bovine A–B heterodimer was labelled with (Eu^3+^) chelate exactly as described previously. Following assay optimization, assay detection range was from 0.02 to 20.0 ng/ml bovine INSL3. Within-assay c.o.v. was <2% across the range and <0.5% in the mid-range, between-assay c.o.v. was consistently <10%. Repeated freeze-thaw cycles as well as experiments to test the effects of storage for different periods at room temperature, 4C or 20C, showed that bovine A–B heterodimeric INSL3 is remarkably stable with >93% recoveries at all times. This assay is highly specific for ruminant INSL3, with ≤0.1% cross-reactivity to human and rat INSL3, and no detectable cross-reactivity to porcine relaxin, IGF1 or insulin.

#### Statistical analysis

All data were analysed employing GraphPad Prism 5.0 software using 2-way ANOVA with Neumann-Keuls post hoc test of significance. Some data were additionally analysed using a standard t test. Differences were considered significant at P<0.05.
